# Supplementary material for: Variations in Antibiotic Use and Sepsis Management in Neonatal Intensive Care Units: A European Survey
Source: Antibiotics (Basel). 2021 Aug 27;10(9):1046. doi: 10.3390/antibiotics10091046 (PMC8469483; doi:10.3390/antibiotics10091046)
Supplement: Supplementary file 1 [file antibiotics-10-01046-s001.zip › Suppl S1.pdf]

Supplemental material S1: List of countries with 6 or less responses to the survey.	
**Country**	**Number of responses (n)**
Albania	1
Austria	6
Belarus	1
Bosnia and Herzegovina	2
Croatia	1
Cyprus	1
Estonia	5
Finland	3
Hungary	4
Iceland	1
Ireland	3
Latvia	2
Lithuania	2
Montenegro	1
North Macedonia	1
Norway	3
Romania	5
Serbia	1
Slovakia	1
Slovenia	1
Switzerland	5
