# Supplementary material for: Variations in Antibiotic Use and Sepsis Management in Neonatal Intensive Care Units: A European Survey
Source: Antibiotics (Basel). 2021 Aug 27;10(9):1046. doi: 10.3390/antibiotics10091046 (PMC8469483; doi:10.3390/antibiotics10091046)
Supplement: Supplementary file 1 [file antibiotics-10-01046-s001.zip › Suppl S2.pdf]

**Supplemental material S2: Antibiotic combinations in early-onset sepsis, late-onset sepsis, and necrotizing enterocolitis.**		
EARLY ONSET SEPSIS	**Number of responses (n)**	**Percent (%)**
AMPI/GENT	148	54.61
PEN/GENT	47	17.34
AMPI/AMIKA	28	10.33
AMPI	8	2.95
PEN/AMIKA	7	2.58
AMPI/TOBRA	5	1.85
AMPI/GENT/AMIKA	3	1.11
GENT/CEFOTA	3	1.11
AMOXI/GENT	2	0.74
AMPI/AMIKA/CEFTA	2	0.74
AMPI/CEFOTA	2	0.74
AMPI/NETIL	2	0.74
PEN/AMPI/GENT	2	0.74
AMIKA/MEROPENEM	1	0.37
AMIKA/PIPE_TAZO	1	0.37
AMOXI/AMIKA	1	0.37
AMPI/CEFURO	1	0.37
AMPI/GENT/CEFOTA	1	0.37
GENT/PIPE_TAZO	1	0.37
MEROPENEM/VANCO	1	0.37
PEN/AMIKA/CEFOTA	1	0.37
PEN/AMINA	1	0.37
PEN/AMPI/CEFOTA	1	0.37
PEN/NETIL	1	0.37
PEN/TOBRA	1	0.37
Total	271	100.00
LATE ONSET SEPSIS	**Number of responses (n)**	**Percent (%)**
AMIKA/VANCO	29	10.70
CEFOTA/VANCO	22	8.12
MEROPENEM/VANCO	21	7.75
AMPI/GENT	16	5.90
GENT/FLUCLO	13	4.80
GENT/VANCO	12	4.43
GENT/CEFOTA/VANCO	10	3.69
PIPE_TAZO	8	2.95
AMIKA/FLUCLO	7	2.58
AMIKA/OXACILLIN	7	2.58
AMPI/CEFOTA	7	2.58
AMIKA	6	2.21
AMIKA/CEFOTA	6	2.21
VANCO/PIPE_TAZO	6	2.21
AMIKA/CEFOTA/VANCO	5	1.85
AMPI/GENT/CEFOTA	5	1.85
CEFTA/VANCO	5	1.85
GENT/CEFOTA	5	1.85
PEN/GENT	5	1.85
AMIKA/TEICO	4	1.48
GENT/OXACILLIN	4	1.48
AMPI/AMIKA	3	1.11
GENT/PIPE_TAZO	3	1.11
PEN/GENT/PIPE_TAZO	3	1.11
VANCO	3	1.11
AMIKA/MEROPENEM	2	0.74
AMIKA/MEROPENEM/VANCO/PIPE_TAZO	2	0.74

AMIKA/PIPE_TAZO	2	0.74
AMPI/CEFOTA/VANCO	2	0.74
CEFOTA	2	0.74
GENT/CEFTA/VANCO	2	0.74
GENT/VANCO/PIPE_TAZO	2	0.74
MEROPENEM	2	0.74
MEROPENEM/VANCO/PIPE_TAZO	2	0.74
TEICO	2	0.74
AMIKA/CEFOPERAZONA	1	0.37
AMIKA/CEFOTA/CEFTA/PIPE_TAZO	1	0.37
AMIKA/CEFOTA/CEFTA/VANCO/PIPE_TAZO	1	0.37
AMIKA/CEFTA	1	0.37
AMIKA/CEFTA/VANCO	1	0.37
AMIKA/GENT/PIPE_TAZO	1	0.37
AMIKA/MEROPENEM/VANCO	1	0.37
AMIKA/VANCO/FLUCLO	1	0.37
AMIKA/VANCO/PIPE_TAZO	1	0.37
AMIKA/VANCO/PIP_TAZO	1	0.37
AMPI/AMIKA/CEFOTA/MEROPENEM/VANCO	1	0.37
AMPI/AMIKA/CEFTA/MEROPENEM/VANCO/ METRONIDAZOLE	1	0.37
AMPI/GENT/CEFTA	1	0.37
AMPI/GENT/MEROPENEM/VANCO/FLUCLO	1	0.37
AMPI_SULBAC/GENT	1	0.37
AMPI_SULBAC/VANCO	1	0.37
CEFOTA/FLUCLO	1	0.37
CEFOTA/MEROPENEM	1	0.37
CEFOTA/TEICO	1	0.37
CEFOTA/VANCO/PIPE_TAZO	1	0.37
CEFTA/FLUCLO	1	0.37
CEFTA/MEROPENEM	1	0.37
CEFTA/TEICO	1	0.37
GENT	1	0.37
GENT/AMIKA/MEROPENEM/VANCO	1	0.37
GENT/CEFOTA/MEROPENEM/PIPE_TAZO	1	0.37
GENT/CEFOTA/MEROPENEM/VANCO	1	0.37
GENT/CEFOTA/VANCO/PIPE_TAZO	1	0.37
GENT/MEROPENEM	1	0.37
GENT/MEROPENEM/VANCO	1	0.37
GENT/TEICO	1	0.37
IMIPENEM	1	0.37
PEN/NETIL	1	0.37
TOBRA/CEFOTA	1	0.37
TOBRA/CEFUROXIME	1	0.37
VANCO/FLUCLO	1	0.37
Total	271	100.00
NECROTIZING ENTEROCOLITIS	**Number of responses (n)**	**Percent (%)**
AMPI/GENT/METRONIDAZOLE	27	10.00
MEROPENEM/VANCO	25	9.26
AMPI/GENT	12	4.44
MEROPENEM/VANCO/METRONIDAZOLE	10	3.70
MEROPENEM	9	3.33
AMIKA/PIPE_TAZO	8	2.96
AMIKA/VANCO/METRONIDAZOLE	8	2.96
CEFOTA/VANCO/METRONIDAZOLE	8	2.96
METRONIDAZOLE	8	2.96
PIPE_TAZO	8	2.96
GENT/AMOXICLAV	7	2.59
VANCO/PIPE_TAZO	7	2.59
GENT/PIPE_TAZO	6	2.22

AMIKA/CEFOTA/METRONIDAZOLE	5	1.85
AMIKA/CEFOTA/VANCO/METRONIDAZOLE	5	1.85
GENT/CEFOTA/VANCO/METRONIDAZOLE	5	1.85
PEN/GENT/METRONIDAZOLE	5	1.85
AMIKA/VANCO/PIPE_TAZO	4	1.48
AMPI/AMIKA/METRONIDAZOLE	4	1.48
GENT/VANCO/METRONIDAZOLE	4	1.48
MEROPENEM/METRONIDAZOLE	4	1.48
AMIKA/AMOXICLAV	3	1.11
AMIKA/GENT/METRONIDAZOLE	3	1.11
AMIKA/VANCO	3	1.11
CEFOTA/METRONIDAZOLE	3	1.11
CEFOTA/VANCO	3	1.11
CEFTA/METRONIDAZOLE	3	1.11
CEFTA/VANCO/METRONIDAZOLE	3	1.11
GENT/CEFOTA/METRONIDAZOLE	3	1.11
METRONIDAZOLE/PIPE_TAZO	3	1.11
AMIKA/CEFOTA	2	0.74
AMIKA/MEROPENEM/METRONIDAZOLE	2	0.74
AMIKA/METRONIDAZOLE	2	0.74
AMIKA/METRONIDAZOLE/PIPE_TAZO	2	0.74
AMPI/GENT/CEFOTA	2	0.74
AMPI/MEROPENEM	2	0.74
GENT/CEFOTA/VANCO	2	0.74
GENT/METRONIDAZOLE/PIPE_TAZO	2	0.74
GENT/VANCO/PIPE_TAZO	2	0.74
MEROPENEM/TEICO	2	0.74
VANCO/METRONIDAZOLE/PIPE_TAZO	2	0.74
AMIKA/CEFTA/MEROPENEM/METRONIDAZOLE	1	0.37
AMIKA/CEFTA/METRONIDAZOLE	1	0.37
AMIKA/CEFTA/ORNIDAZOLE	1	0.37
AMIKA/CEFTA/TEICO	1	0.37
AMIKA/MEROPENEM	1	0.37
AMIKA/METRONIDAZOLE/FLUCLO	1	0.37
AMIKA/METRONIDAZOLE/TEICO	1	0.37
AMPI	1	0.37
AMPI/AMIKA	1	0.37
AMPI/GENT/METRONIDAZOLE	1	0.37
AMPI/GENT/CEFOTA/METRONIDAZOLE	1	0.37
AMPI/GENT/CEFOTA/VANCO/METRONIDAZOLE	1	0.37
AMPI/GENT/CLINDA	1	0.37
AMPI/TOBRA/METRONIDAZOLE	1	0.37
CEFOPERAZONE/AMIKA/METRONIDAZOLE	1	0.37
CEFOPERAZONE/VANCO	1	0.37
CEFOTA/MEROPENEM	1	0.37
CEFOTA/VANCO/ORNIDAZOLE	1	0.37
CEFTA/METRONIDAZOLE/TEICO	1	0.37
CEFTA/VANCO	1	0.37
GENT/AMIKA/MEROPENEM	1	0.37
GENT/AMIKA/VANCO	1	0.37
GENT/AMPI	1	0.37
GENT/AMPI_SULBACTAM	1	0.37
GENT/CEFOTA/MEROPENEM/VANCO/METRONIDAZOLE	1	0.37
GENT/CEFTA/VANCO/METRONIDAZOLE	1	0.37
GENT/MEROPENEM	1	0.37
GENT/MEROPENEM/VANCO	1	0.37
GENT/METRONIDAZOLE	1	0.37
GENT/METRONIDAZOLE/FLUCLO	1	0.37
GENT/METRONIDAZOLE/TEICO	1	0.37
GENT/VANCO/PIPE_TAZO	1	0.37
GENT/VANCO	1	0.37
MEROPENEM/VANCO	1	0.37

MEROPENEM/VANCO/METRONIDAZOLE/PIPE_TAZO	1	0.37
METRONIDAZOLE/CLINDAMYCIN	1	0.37
PEN/AMPI/METRONIDAZOLE	1	0.37
TOBRA/CEFTA/VANCO/METRONIDAZOLE	1	0.37
TOBRA/MEROPENEM/METRONIDAZOLE	1	0.37
TOBRA/PIPE_TAZO	1	0.37
VANCO/METRONIDAZOLE	1	0.37
VANCO/NETILMICIN/METRONIDAZOLE	1	0.37
Total	270	100.00

AMIKA: amikacin; AMPI: ampicillin; CEFOTA: cefotaxime; CEFTA: ceftazidime; FLUCLO: flucloxacillin; GENT: gentamicin; MEROPENEM: meropenem; METRONIDAZOLE: metronidazole; OXACILLIN: oxacillin; PEN: penicillin; PIPE_TAZO: piperacillin-tazobactam; TEICO: teicoplanin; TOBRA: tobramycin; VANCO: vancomycin.
