# Supplementary material for: Variations in Antibiotic Use and Sepsis Management in Neonatal Intensive Care Units: A European Survey
Source: Antibiotics (Basel). 2021 Aug 27;10(9):1046. doi: 10.3390/antibiotics10091046 (PMC8469483; doi:10.3390/antibiotics10091046)
Supplement: Supplementary file 1 [file antibiotics-10-01046-s001.zip › Suppl S3.pdf]

**Supplemental material S3: Biological markers and laboratory tests used to diagnose neonatal sepsis and decide to suspend antibiotic treatment.**		
**A. Start Antibiotics**		
**Tests**	**Number of responses**	**Percent (%)**
CRP/BC	72	26,6
CRP/PRO/BC	66	24,4
CRP/PRO/PCR/BC	18	6,6
CRP/BC/FBC	15	5,5
CRP/CYTO/BC	11	4,1
CRP/PCR/BC	10	3,7
CRP/PRO	8	3,0
CRP/PRO/CYTO/BC	7	2,6
CRP/BC/HERO	6	2,2
CRP/PRO/BC/FBC	5	1,8
CRP/PRO/BC/HERO	5	1,8
BC	4	1,5
CRP/PRO/DNA/BC	4	1,5
CRP/CYTO	3	1,1
CRP/CYTO/PCR/BC	3	1,1
CRP/PRO/CYTO/PCR/BC	3	1,1
CRP	2	0,7
CRP/BC/HERO/FBC	2	0,7
CRP/CYTO/PCR/DNA/BC	2	0,7
CRP/FBC	2	0,7
CRP/PRO/BC/HERO/FBC	2	0,7
CRP/PRO/CYTO/PCR/BC/HERO	2	0,7
CRP/PRO/PCR	2	0,7
CRP/PRO/PCR/BC/FBC	2	0,7
CRP/PRO/PCR/DNA/BC	2	0,7
CEP/PRO/BC	1	0,4
CRP/CYTO/BC/FBC	1	0,4
CRP/CYTO/DNA/BC	1	0,4
CRP/DNA/BC/HERO	1	0,4
CRP/PCR/BC/FBC	1	0,4
CRP/PRO/BC/FBC/CSF/UC	1	0,4
CRP/PRO/CYTO/BC/HERO	1	0,4
CRP/PRO/CYTO/DNA/BC	1	0,4
CRP/PRO/CYTO/DNA/BC/HERO	1	0,4
CRP/PRO/FBC	1	0,4
PCR/BC	1	0,4
PRO/BC	1	0,4
PRO/BC/FBC	1	0,4
**Total**	**271**	**100,0**
**B. Stop Antibiotics**		

Tests	Number of responses	Percent (%)
CRP/BC	112	41,3
CRP/PRO/BC	36	13,3
CRP	32	11,8
BC	22	8,1
CRP/PRO	17	6,3
CRP/PCR/BC	7	2,6
CRP/BC/FBC	6	2,2
CRP/FBC	3	1,1
CRP/PRO/BC/HERO	3	1,1
NONE	3	1,1
PRO/BC	3	1,1
CRP/BC/HERO	2	0,7
CRP/CYTO	2	0,7
CRP/PRO/BC/FBC	2	0,7
CRP/PRO/BC/HERO/FBC	2	0,7
CRP/PRO/CYTO/BC	2	0,7
CRP/PRO/DNA/BC	2	0,7
CRP/PRO/PCR	2	0,7
CRP/PRO/PCR/BC	2	0,7
PCR/DNA/BC	2	0,7
CRP/CYTO/BC	1	0,4
CRP/CYTO/FBC	1	0,4
CRP/CYTO/PCR/BC	1	0,4
CRP/DNA/BC/HERO	1	0,4
CRP/PRO/FBC	1	0,4
CRP/PRO/PCR/DNA/BC	1	0,4
PCR/BC	1	0,4
PRO	1	0,4
PRO/CYTO/HERO	1	0,4
**Total**	**271**	**100,0**

BC: blood cultures; CRP: C reactive protein; CYTO: cytokines; DNA: DNA microarray-based methods; FBC: full blood count; HERO: HeRo monitoring; PCR: PCR based methods; PRO: procalcitonin.
