# Supplementary material for: Variations in Antibiotic Use and Sepsis Management in Neonatal Intensive Care Units: A European Survey
Source: Antibiotics (Basel). 2021 Aug 27;10(9):1046. doi: 10.3390/antibiotics10091046 (PMC8469483; doi:10.3390/antibiotics10091046)
Supplement: Supplementary file 1 [file antibiotics-10-01046-s001.zip › Suppl S4.pdf]

Supplemental material S4. Maternal policy screening for Group B Streptococcus (GBS)

A. Does your hospital screen ALL mothers for Group B Streptococcus (GBS) colonization at 35-37 weeks of gestation?: **NO**

Country	Number of responses (n)	Percent (%)
Turkey	8	16,0
Netherlands	6	12,0
Germany	5	10,0
Sweden	5	10,0
Denmark	4	8,0
Israel	4	8,0
Russia	4	8,0
United Kingdom	3	6,0
Greece	2	4,0
Ireland	2	4,0
Austria	1	2,0
Czech Republic	1	2,0
Finland	1	2,0
France	1	2,0
Iceland	1	2,0
Poland	1	2,0
Romania	1	2,0
Total	50	100,0

B. Does your hospital screen ALL mothers for Group B Streptococcus (GBS) colonization at 35-37 weeks of gestation?: **ONLY FOR SELECTED MOTHERS**

Country	Number of responses (n)	Percent (%)
Bulgaria	6	10,5
Sweden	6	10,5
Greece	5	8,8
Estonia	4	7,0
Israel	4	7,0
Netherlands	4	7,0
Romania	4	7,0
Russia	3	5,3
Turkey	3	5,3
United Kingdom	3	5,3
Denmark	2	3,5
Norway	2	3,5
Albania	1	1,8
Austria	1	1,8
Belarus	1	1,8
Bosnia and Herzegovina	1	1,8

Cyprus	1	1,8
France	1	1,8
Germany	1	1,8
Hungary	1	1,8
Ireland	1	1,8
Portugal	1	1,8
Serbia	1	1,8
Total	57	100,0

C. Does your hospital screen ALL mothers for Group B Streptococcus (GBS) colonization at 35-37 weeks of gestation?: **YES**

**Country**	**Number of responses (n)**	**Percent (%)**
Italy	41	25,0
France	19	11,6
Belgium	17	10,4
Portugal	15	9,1
Spain	14	8,5
Poland	11	6,7
Czech Republic	8	4,9
Switzerland	5	3,0
Austria	4	2,4
Germany	4	2,4
Denmark	3	1,8
Hungary	3	1,8
Bulgaria	2	1,2
Finland	2	1,2
Latvia	2	1,2
Lithuania	2	1,2
Bosnia and Herzegovina	1	,6
Croatia	1	,6
Estonia	1	,6
Greece	1	,6
Montenegro	1	,6
Netherlands	1	,6
North Macedonia	1	,6
Norway	1	,6
Russia	1	,6
Slovakia	1	,6
Slovenia	1	,6
United Kingdom	1	,6
Total	164	100,0
