# Supplementary material for: Variations in Antibiotic Use and Sepsis Management in Neonatal Intensive Care Units: A European Survey
Source: Antibiotics (Basel). 2021 Aug 27;10(9):1046. doi: 10.3390/antibiotics10091046 (PMC8469483; doi:10.3390/antibiotics10091046)
Supplement: Supplementary file 1 [file antibiotics-10-01046-s001.zip › Suppl S5.pdf]

**Supplemental material S5. Summary of questionnaire**		
Q1	In which country is your hospital located?	Country
Q2	How would you classify the level of care in your neonatology unit?	Level 1 Neonatal Unit (look after healthy newborns but are relatively stable and mature)<br>Level 2 Neonatal Unit (look after babies who need more advanced support like TPN, CPAP, brief CMV...)<br>Level 3 NICU (look after the most premature and unwell babies)<br>Level 3 NICU with neonatal surgery, cardiac surgery or neurosurgery<br>Mixed pediatric and neonatal intensive care unit
Q4	In your unit, do you care for premature newborns under 28 weeks until discharge?	Yes<br>No
Q5	How many NEONATAL ADMISSIONS of babies did you have in 2020? (in numbers please)	Number
Q6	How many ADMISSIONS of babies < 1500 gr did you have in 2020?	Less than 30<br>Between 30 and 50<br>More than 50
Q7	How many intensive care beds / incubators do you have in your unit? (in numbers please)	Number
Q8	Does your neonatal unit has a written antibiotic GUIDELINE to treat empirically EARLY ONSET NEONATAL SEPSIS?	Yes<br>No
Q9	If your answer to the previous question is YES, how would you describe the level of adherence to the GUIDELINE?	Very low<br>Low<br>Intermediate<br>High<br>Very high
Q10	Does your neonatal unit has a written antibiotic GUIDELINE to treat empirically LATE ONSET NEONATAL SEPSIS?	Yes<br>No
Q11	If your answer to the previous question is YES, how would you describe the level of adherence to the GUIDELINE?	Very low<br>Low<br>Intermediate<br>High<br>Very high
Q12	Does your neonatal unit has a written antibiotic GUIDELINE to STOP treatment in NEONATAL SEPSIS?	Yes<br>No
Q13	If your answer to the previous question is YES, how would you describe the level of adherence to the GUIDELINE?	Very low<br>Low<br>Intermediate<br>High<br>Very high
Q14	Does your MATERNITY unit has a written antibiotic stewardship to IDENTIFY and TREAT MOTHERS with risk of perinatal infection?	Yes<br>No
Q15	If your answer to the previous question is YES, how would you describe the level of adherence to the GUIDELINE?	Very low<br>Low<br>Intermediate<br>High<br>Very high
Q16	Does your hospital screen ALL mothers for Group B Streptococcus (GBS) colonization at 35-37 weeks of gestation?	Yes<br>No<br>Only for selected mothers
Q17	Does your neonatal unit uses any tool (like Kaiser Permanente Early Onset Sepsis) to predict the risk of microbiologically confirmed	Yes<br>No

	infection in order to reduce unnecessary investigations and antibiotic therapy with some success?	Only sometimes, depending on the neonatologist
Q18	Which is your initial EMPIRIC therapy for EARLY ONSET NEONATAL SEPSIS? Could you specify the dose regime for the selected antibiotics in the last text box?	Penicillin<br>Ampicillin<br>Gentamicin<br>Tobramycin<br>Amikacin<br>Cefotaxime<br>Ceftazidime<br>Meropenem<br>Vancomycin<br>Nafcillin<br>Metronidazole<br>Piperacillin-tazobactam<br>Teicoplanin<br>Flucloxacillin<br>Oxacillin<br>Other (please specify)
Q19	Which is your initial EMPIRIC therapy for LATE ONSET NEONATAL SEPSIS? Could you specify the dose regime for the selected antibiotics in the last text box?	Penicillin<br>Ampicillin<br>Gentamicin<br>Tobramycin<br>Amikacin<br>Cefotaxime<br>Ceftazidime<br>Meropenem<br>Vancomycin<br>Nafcillin<br>Metronidazole<br>Piperacillin-tazobactam<br>Teicoplanin<br>Flucloxacillin<br>Oxacillin<br>Other (please specify)
Q20	Which is your initial EMPIRIC therapy for babies with Necrotizing Enterocolitis (NEC)?	Penicillin<br>Ampicillin<br>Gentamicin<br>Tobramycin<br>Amikacin<br>Cefotaxime<br>Ceftazidime<br>Meropenem<br>Vancomycin<br>Nafcillin<br>Metronidazole<br>Piperacillin-tazobactam<br>Teicoplanin<br>Flucloxacillin<br>Other (please specify)
Q21	Does your Neonatal Unit perform TDM (Therapeutic Drug Monitoring) as per local/national guidelines, always that you prescribe...?	Gentamycin<br>Tobramycin<br>Vancomycin<br>Teicoplanin<br>Amikacin<br>Never<br>Other (please specify)

Q22	Which markers do you routinely use in your neonatal unit for the DIAGNOSIS and MONITORING of neonatal infections?	C reactive protein<br>Procalcitonin<br>Cytokines<br>PCR based methods<br>DNA microarray-based methods<br>Blood culture<br>HeRo monitoring (heart rate variability monitoring)<br>Other (please specify)
Q23	Which markers do you really routinely use in your NICU to STOP your antibiotics in neonatal infections?	C reactive protein<br>Procalcitonin<br>Cytokines<br>PCR based methods<br>DNA microarray-based methods<br>Blood culture<br>HeRo monitoring (heart rate variability monitoring)<br>Other (please specify)
Q24	What is your usual practice regarding the decision to perform a lumbar puncture and analyze CSF (cerebrospinal fluid) in a CLINICALLY SEPTIC newborn?	I always perform a lumbar puncture if the newborn is clinically stable<br>I decide to perform a lumbar puncture, based on the patients' history, risk factors, clinical features...<br>I decide to perform a lumbar puncture depending on blood markers (CRP, for example)<br>I decide to perform a lumbar puncture depending on the positivity or negativity of the blood culture<br>Other (please specify)
Q25	Do you use ORAL antibiotics in newborns admitted in your NICU?	Yes<br>No
Q26	If your answer is YES to the previous question, could you explain in which clinical situation you would use them?	In case of limited vascular access<br>As a prophylactic treatment for babies at risk of infection<br>As an optional treatment for mild infections<br>Other (please specify)
Q27	Do you use FUNGAL prophylaxis in your VLBW preterm babies?	Yes<br>No
Q28	Which regimen of IV FLUCONAZOLE do you normally use as prophylaxis in your preterm babies?	Free text
Q29	Do you normally swap to ORAL NYSTATIN when your preterm baby is on MINIMAL ENTERAL FEEDING? If not, when?	Free text
