# Supplementary material for: Variations in Antibiotic Use and Sepsis Management in Neonatal Intensive Care Units: A European Survey
Source: Antibiotics (Basel). 2021 Aug 27;10(9):1046. doi: 10.3390/antibiotics10091046 (PMC8469483; doi:10.3390/antibiotics10091046)
Supplement: Supplementary file 1 [file antibiotics-10-01046-s001.zip › Suppl S6.pdf]

Supplementary material S6: List of participants in the survey. Only those participants who wished to provide their names and affiliation data have been collected in this list (European Antibiotics Study Group - EASG)

Agneta Golan, Soroka Medical Center Israel (Israel)
Agnieszka Kordek, Pomeranian Medical University in Szczecin, Szczecin (Poland)
Agustín Molina Merino, Valencia University Clinical Hospital, Valencia (Spain)
Alessandra Vittorini, Macedonio Melloni Hospital, Milan (Italy)
Alexandre Lapillonne, Necker Enfants Malades Hospital, Paris (France)
Alexandrina Portela, Pedro Hispano Hospital (Portugal)
Almerinda Barroso Pereira, Hospital of Braga (Portugal)
Andreas Ohlin, Örebro University Hospital, Örebro (Sweden)
Andreas Trobisch, Medical University of Graz (Austria)
Andreia Teles, Vila Nova Gaia/Espinho Hospital Center (Portugal)
Angeliki G Kontou , Hippokration general hospital of Thessaloniki, hessaloniki (Greece)
Anja Stein, Essen University Hospital, Essen (Germany)
Anna Daskalaki, Attikon University Hospital, Athens (Greece)
Anne Smits, University Hospitals Leuven, Leuven (Belgium)
Annika Tiit-Vesingi, East-Tallinn Central Hospital, Tallinn (Estonia)
Anselmo Costa, Garcia de Orta Hospital, Almada (Portugal)
Anton Yuditskiy, Izhevsk State Medical Academy, Izhevsk (Russia)
Antonio Cuñarro, Alcorcón Foundation University Hospital, Madrid (Spain)
Artem Chernykh, Kuzbass Regional Children Clinical Hospital (Russia)
Aslı Memisoglu, Marmara University (Turkey)
Aurélie Cantais, North Saint Etienne University Hospital Center, Saint Eienne (France)
Barbara Królak-Olejnik, Wrocław Medical University, Wrocław (Poland)
Belén Fernández Colomer, Central University Hospital of Asturias, Oviedo (Spain)
Benny Bar-Oz, Hadassah and Hebrew University Medical centers, Jerusalem (Israel)
Benoit Boeuf, University Hospital of Clermont Ferrand (France)
Bernhard Resch, Medical University of Graz (Austria)
Camilla Gizzi, Sandro Pertini Hospital, Rome (Italy)
Carlo Dani, Careggi University Hospital of Florence, Florence (Italy)
Carmen Carvalho, North Mother and Child Center, Porto (Portugal)
Carmen Rodríguez, Children's Hospital, Civil Hospital of Brescia, Brescia (Italy)
Cesare Arioni, Policlinico San Martino Hospital, Genova (Italy)
Chris H.P. van den Akker, Amsterdam UMC, Amsterdam (The Netherlands)
Christoph Binder, Medical University of Vienna, Vienna (Austria)
Claudio Migliori, San Giuseppe Hospital, Milan (Italy)
Colm O'Donnell, National Maternity Hospital and University College, Dublin (Ireland)
Cristina Haass, San Pietro FBF Hospital, Rome (Italy)
Cristina Tuoni, Santa Chiara Hospital, Pisa (Italy)
D Mitanchez, Bretonneau University Hospital Center Tours (France)
Dan Waisman, Carmel Medical Center and Faculty of Medicine - Technion IIT, Haifa (Israel)
Daniel Vijlbrief, Wilhelmina children's hospital, UMC Utrecht, Utrecht (The Netherlands)
Danila Mazzeo, AOU Policlinico Di Messina, Messina (Italy)
Darinka Šumanović-Glamuzina, University Hospital Mostar, Clinic for Child diseases, Mostar (Bosnia and Herzegovina)
Dilek Kahvecioğlu, Universty of Health Sciences Ankara Training and Research Hospital, Ankara (Turkey)
Dimitrioska R an Al Khalili D, University Clinic for obstetric and gynecology, Skopje (North Macedonia)
Dusek J., Ceske Budejovice Hospital (Czech Republic)

Dzmitry Sankovec, National Research and Practical Center Mother and Child (Belarus)
E. de Kort, Maxima Medical Center Veldhoven (The Netherlands)
Elisa Mojica Muñoz, Nykobing Falster Hospital (Denmark)
Elisabeth Henrion, Namur Regional Hospital, Namur (Belgium)
Emmanuelle Motte-Signoret, Saint-Germain-en-Laye Intercommunal Hospital Center, Poissy (France)
Envera Lekic, University Clinical Center , Institute For Children Diseases, Center of Neonatology, Podgorica (Montenegro)
Eric Cavatorta, Marie Curie Civil Hospital, Charleroi University Hospital Center (Belgium)
Eric Giannoni, Lausanne University Hospital, Lausanne (Switzerland)
Erik Normann, Department of Women's and Children's Health, Uppsala University Children's Hospital (Sweden)
Eugene Dempsey, Cork University Maternity Hospital, Cork (Ireland)
Federico Zaglia, Mater Salutis General Hospital, Legnago, Verona (Italy)
Femi Adeniyi, Arrowe Park Hospital, Wirral (United Kingdom)
Fernanda Melo, Beatriz Angelo Hospital, Loures (Portugal)
Francesco Cardona, Medical University Vienna (Austria)
Francesco Pullano, San Giugliano Di Giugliano Hospital (Italy)
Francesco Raimondi, Federido II Napoly University (Italy)
G. Delanghe, ZNA Middelheim, Antwerp (Belgium)
G. Maffei and G. Rinaldi, Foggia Reunited Hospital (Italy)
G. Ohia, Provincial Specialist Hospital in Wroclaw, Wroclaw (Poland)
Gabriel Dimitriou, General University Hospital of Patras (Greece)
Georgios Mavrogeorgos, Aglaia Kyriakou Children's Hospital, Athens (Greece)
Georgios Papadopoulos, Alexandras Maternity Hospital, Athens (Greece)
Gholamreza Dayani, Zeeland University Hopistal, Roskilde (Denmark)
Giuseppe Latorre, Ente Ecclesiastico Miulli Hospital, Acquaviva delle Fonti (Italy)
Grażyna Pazera, Regional Hospital in Kielce, Kielce (Poland)
H. Gözde Kanmaz Kutman, Ankara Bilkent City Hospital, Ankara (Turkey)
H.R. Taal, Erasmus Medical Center, Rotterdam (The Netherlands)
Hallvard Reigstad, Haukeland University Hospital, Bergen (Norway)
Hana Wiedermannová, University Hospital Ostrava (Czech Republic)
Hasenöhrlová Kristýna, Pardubice Hospital (Czech Republic)
Henrique Soares, São João Universitary Hospital Center, Porto (Portugal)
Ignacio Benítez-Segura, Son Espases University Hospital, Palma de Mallorca (Spain)
Ilaria Lori, San Giovanni di Dio Hospital, Firenze (Italy)
J. De Buyst, Tivoli University Hospital Center (Belgium)
Jan Maly, Charles University Hospital, Hradec Kralove (Czech Republic)
Jean-Michel Hascoët, Regional University Maternity Hospital - Nancy CHRU, Nancy (France)
Joanna Kasprzycka, E.Biernacki Obstetric and Gynecology Hospital, Walbrzyc (Poland)
Joanna Seliga-Siwecka, Princess Anna Mazowiecka Hospital, Warsaw (Poland)
Jolanta Wroblewska, Norrlands University Hospital, Umeå (Sweden)
Juan Calviño, Tras-os-Montes e Alto Douro Hospitalary Center, Vila Real (Portugal)
Juliane Tautz, University Hospital Düsseldorf, Düsseldorf (Germany)
Katleen Plaskie, GZA Hospital Sint Augustinus, Antwerp (Belgium)
Laura Fazilleau, University Hospital Center Côte de Nacre, Caen (France)
Laura Sanchez García, La Paz University Hospital, Madrid (Spain)
Leonid Pankratov, Children's Hospital number 1, St. Petesburg (Russia)
Lili Mikecin, Trbovlje General Hospital (Slovenia)
Liliya Vakrilova, University Maternity Hospital "Maichin dom", Sofia (Bulgaria)
Linda Vad Pedersen, Aarhus University Hospital, Aarhus (Denmark)
Lise Aunsholt, Rigshospitalet, Copenhague (Denmark)

Livica Fratiman, Constanta County Emergency Clinical Hospital, Constanta (Romania)
Ludo Mahieu, University Hospital Antwerp (Belgium)
M. Tirantello, Umberto I Hospital, Siracusa (Italy)
M.Chvilova Weberova, Havlickuv Brod Hospital (Czech republic)
Magdalena Rutkowska, Institute of Mother and Child, Warsaw (Poland)
Malgorzata Czyzewska, University Clinical Hospital in Wroclaw (Poland)
Marcelino Pumarada Prieto, Álvaro Cunqueiro Hospital, Vigo (Spain)
Marcio Accioly Sippel Fossari, Santa Luiza Maternity (Brazil)
Maria Livia Ognean, County Clinical Emergency Hospital of Sibiu (Romania)
Maria Pierro, Mauricio Bufalini Hospital, Cesena (Italy)
Maria Stamatin, Cuza-Voda Hospital of Obstetrics and Gynecology (Romania)
Marie Antoinette Frick, Vall d'Hebron Hospital, Barcelona (Spain)
Mari-Liis Ilmoja, Tallinn Children`s Hospital, Tallinn (Estonia)
Marine Butin, Civils Hospices of Lyon, Lyon (France)
Mario Barbarini, St. Anna Hospital, Como (Italy)
Mario Giuffrè, University of Palermo, Palermo (Italy)
Mark Dzietko, University of Essen (Germany)
Martin Matas, University hospital in Pilsen (Czech Republic)
Martin Weissensteiner, Clinic for Neonatology, Kepler University Hospital, Linz (Austria)
Mathias Urs Gebauer, Children's Clinic, Biel/Bienne Hospital Center (Switzerland)
Matilde Ciccia, Maggiore Hospital, Bologna (Italy)
Matthias C. Hütten, Neonatology Maastricht University Medical Center, Maastricht (The Netherlands)
Michelangelo Barbaglia, "Giuseppe Castelli" Hospital of Verbania-Pallanza (Italy)
Míriam Martínez Gómez, Quironsalud Valencia Hospital, Valencia (Spain)
Mirjam van Weissenbruch, VU University Medical Center, Amsterdam (The Netherlands)
Mondì Vito, Policlinic Casilino Hospital, Rome (Italy)
Muriel Ferreira and Dolores Faria, Coimbra Hospital and University Center, Dr. Bissaya Barreto Maternity Hospital, Coimbra (Portugal)
Nadya Yousef, Antoine Béclère Medical center, Greater Paris University Hospitals (France)
Neli Jekova, University Maternity Hospital "Maichin dom", Sofia (Bulgaria)
Nicoletta Mainini and Elena Priante, Padua University Hospital, Padua (Italy)
Olayinka Kowobari, Royal Stoke University Hospital, Stoke-on-Trent (United Kingdom)
Olga Ksenofontova, Yekaterinburg Clinical Perinatal Center (Russia)
Olga Voutsen, Professor Doutor Fernando Fonseca Hospital, Amadora (Portugal)
Omer Erdeve, Ankara University Children's Hospital, Ankara (Turkey)
Paola Lago, Cà Foncello Hospital, Treviso (Italy)
Paola Sikias, Antony Private Hospital - Ramsay Santé, Antony (France)
Pascal Bolieau, Saint-Germain-en-Laye Intercommunal Hospital Center, Poissy (France)
Patrick Pladys, University Hospital Center Rennes - Pontchaillou (France)
Pernille Kure, Viborg Regional Hospital, Viborg (Denmark)
Petronilla Meo , Barone Romeo Hospital, Patti, Messina (Italy)
Pia Härkin, Oulu University Hospital, Oulu (Finland)
Pierre Kuhn, Strasbourg University Hospital, Strasbourg (France)
Pierre-Louis Leger, Armand Trousseau Hospital (France)
Pierre-Yves Wachter, Saint-Germain-en-Laye Intercommunal Hospital Center, Poissy (France)
Pietro D'Amico, Canizzaro Emergency Hospital, Catania (Italy)
Roberto Bellù, Alessandro Manzoni Hospital, Lecco (Italy)
Roser Porta, Germans Trias i Pujol Hospital, Badalona (Spain)
Rustam Mukhametshin, Regional children's Clinical Hospital, Yekaterinburg (Russia)
Ruth Gottstein, St. Mary's Hospital, Manchester Foundation Trust, Manchester (United Kingdom)
Ryszard Lauterbach, Kraków University Hospital, Kraków (Poland)

Samuli Rautava, Helsinki University Hospital, Helsinki (Finland)
Sandra Salas, Castellón General Universitary Hospital, Castellón (Spain)
Seda Yilmaz Semerci, Istanbul Kanuni Sultan Suleyman Training and Research Hospital, Istanbul (Turkey)
Senem Alkan Özdemir, Izmir, Dr. Behcet Uz Child Disease and Pediatric Surgery Training and Research Hospital (Turkey)
Sergio Eleni Dit Trolli, Nice University Hospital Center, Nice (France)
Serif Hamitoglu, Cukurova University (Turkey)
Shalini Ojha, Royal Derby Hospital, Derby (United Kingdom)
Shmuel Arnon, Meir Medical Center, Kfar Saba (Israel)
Shteryu Boyadzhiev, Lozenetz University Hospital, Sofia (Bulgaria)
Smadar Eventov Friedman, Hadassah Hebrew University Medical Center, Jerusalem (Israel)
Sonia Zangara, Rivoli Hospital, Local Health Company TO 3, Rivoli (Italy)
Sophie Vanhaesebrouck, Ghent University Hospital, Ghent (Belgium)
Stamatia Dagkli, Elena Venizelou Hospital, Athens (Greece)
Stefania Vedovato, San Bortolo Hospital of Vicenza (Italy)
Teresa Hernández, Cabueñes Universitary Hospital, Cabueñes (Spain)
Teresa Tome, Dr Alfredo Da Costa Maternity Hospital, Lisboa (Portugal)
Thordur Thorkelsson, Children's Hospital Iceland, Reykjavik (Iceland)
Tomas Juren, University Hospital Brno (Czech Republic)
Tomas Juren, University Hospital Brno, Brno-Bohunice-Brno-Starý Lískovec (Czech Republic)
Tugba Erener Ercan, Maltepe University Hospital, Maltepe (Turkey)
Tuuli Metsvaht, Tartu University Hospital, Tartu (Estonia)
Tzipi Strauss, Sheba Medical center, Ramat Gan (Israel)
Valerie Biran, University Hospital Center Robert Debré, Paris (France)
Vasiliki Falaina, General Hospital of Nikaia "Agios Panteleimon" (Greece)
Vilma Ivanauskienė, Hospital of Lithuanian University of Health Sciences Kaunas Clinics (Lithuania)
Vincent Rigo, University of Liege, CHU and CHR Liege (Belgium)
Vladan Milovanov, Hospital of Southwester Jutland, Esbjerg, (Denmark)
Wisam S Muhsen, Plymouth University Hospital, Plymouth (United Kingdom)
Xavier Durrmeyer, Intercommunal Hospital of Créteil, Créteil (France)
Zbynek Stranak, Institute for the Care of Mother and Child (Czech Republic)
Zeynep Alp Unkar, Istanbul University, Cerrahpasa Medical School (Turkey)
